# Single-cell Sequencing Reveals Clearance of Blastula Chromosomal Mosaicism in *In Vitro* Fertilization Babies

**DOI:** 10.1016/j.gpb.2022.07.004

**Published:** 2022-08-06

**Authors:** Yuan Gao, Jinning Zhang, Zhenyu Liu, Shuyue Qi, Xinmeng Guo, Hui Wang, Yanfei Cheng, Shuang Tian, Minyue Ma, Hongmei Peng, Lu Wen, Fuchou Tang, Yuanqing Yao

**Affiliations:** 1 Biomedical Pioneering Innovation Center, School of Life Sciences, Peking University, Beijing 100871, China; 2 Department of Gynaecology and Obstetrics, The First Medical Center of PLA General Hospital, Beijing 100853, China; 3 Peking-Tsinghua Center for Life Sciences, Academy for Advanced Interdisciplinary Studies, Peking University, Beijing 100871, China; 4 Reproductive Medicine and Prenatal Diagnosis Center, The University of Hong Kong–Shenzhen Hospital, Shenzhen 518053, China; 5 Beijing Advanced Innovation Center for Genomics, MOE Key Laboratory of Cell Proliferation and Differentiation, Beijing 100871, China

**Keywords:** Mosaicism, Pre-implantation genetic testing for aneuploidy, Next-generation sequencing, Single-cell multi-omics sequencing, Mosaic embryo transfer

## Abstract

Although chromosomal mosaic embryos detected by trophectoderm (TE) biopsy offer healthy embryos available for transfer, high-resolution postnatal karyotyping and chromosome testing of the transferred embryos are insufficient. Here, we applied **single-cell multi-omics sequencing** for seven infants with blastula chromosomal **mosaicism** detected by TE biopsy. The chromosome ploidy was examined by single-cell genome analysis, with the cellular identity being identified by single-cell transcriptome analysis. A total of 1616 peripheral leukocytes from seven infants with embryonic chromosomal mosaicism and three control ones with euploid TE biopsy were analyzed. A small number of blood cells showed copy number alterations (CNAs) on seemingly random locations at a frequency of 0%−2.5% per infant. However, none of the cells showed CNAs that were the same as those of the corresponding TE biopsies. The blastula chromosomal mosaicism may be fully self-corrected, probably through the selective loss of the aneuploid cells during development, and the transferred embryos can be born as euploid infants without mosaic CNAs corresponding to the TE biopsies. The results provide a new reference for the evaluations of transferring chromosomal mosaic embryos in certain situations.

## Introduction

Embryonic mosaicism has been more frequently reported in recent years since the application of new technologies such as next-generation sequencing (NGS) to pre-implantation genetic testing (PGT). Studies have reported that pre-implantation embryos have a high proportion of mosaicism [1], and the rate of diagnosed mosaicism in trophectoderm (TE) varies from 3% to 26.4% [2], [3], [4], [5]. Chromosomal mosaicism refers to the presence of two or more different cell lineages with different ploidies in an individual, which is common during early embryonic development and is caused by segregation errors during mitosis. This leads to the existence of new classification categories in addition to diploids, monosomies, and trisomies, *i.e.*, the presence of intermediate levels. It is also consistent with the mosaic phenomenon and indicates that both euploid and aneuploid cells co-exist in the same embryo.

Among natural pregnancies, mosaics can cause problems such as intrauterine growth retardation or placental insufficiency [6], [7]. Among *in vitro* fertilization (IVF) embryos, the pregnancy rates and live birth rates of the mosaic embryos have been reported to be lower than those of normal embryos [1], [8], [9], [10]. Although the effect of mosaics on the developmental potential of embryos is unknown, mosaic embryos have been reported to be able to develop into live births and even normal babies [11], [12], [13], [14]. Pregnancy and live birth rates in mosaic embryo transfer range from 16% to 47% [1], [2], [11], [13], [14], [15], [16], [17], though the rate is lower in embryos that have full chromosome mosaics or three or more mosaics [1], [8], [10]. Greco et al. [11] first reported clinical results after the implantation of mosaic embryos, and the transplantation of 18 mosaic embryos resulted in 6 full-term babies. Karyotype analysis by chorionic villus sampling (CVS) showed normal karyotype in all neonates.

Although knowing the consequence of embryonic chromosomal mosaicism is important for both IVF pregnancy and natural conception, postnatal evaluation of transferred mosaic embryos is not sufficient. The current evaluation is mainly focused on the rates of pregnancy and birth, while data on single-cell resolution postnatal karyotyping and chromosome testing are still lacking. Here we performed single-cell multi-omics sequencing for peripheral white blood cells obtained from seven infants with embryonic chromosomal mosaicism to explore their chromosome ploidy status at single-cell resolution.

## Results

### Study outline

**Figure 1:**
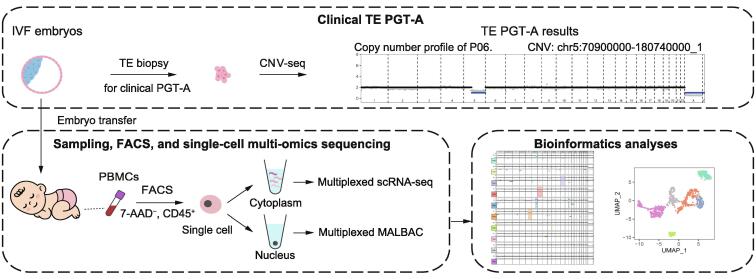
**Study design** Schematic diagram of the workflow of sample collection, sequencing, and data processing. IVF, *in vitro* fertilization; TE, trophectoderm; PGT-A, pre-implantation genetic testing for aneuploidy; CNV-seq, copy number variation sequencing; PBMC, peripheral blood mononuclear cell; FACS, fluorescent activation cell sorting; scRNA-seq, single-cell RNA sequencing; MALBAC, multiple annealing and looping-based amplification cycle.

These seven infants are normally born and grow healthy despite showing chromosomal mosaicism in TE biopsies. We collected trace peripheral blood from these infants and sorted leukocytes by fluorescent activation cell sorting (FACS). Then we performed single-cell multi-omics sequencing that simultaneously obtained genome and transcriptome information of the individual cell (Figure 1). The genome and transcriptome information revealed copy number alterations (CNAs) and the cell identity, respectively. Details of these methods are in the Materials and methods.

### Chromosome ploidy in mosaic blastocyst infants

**Figure 2:**
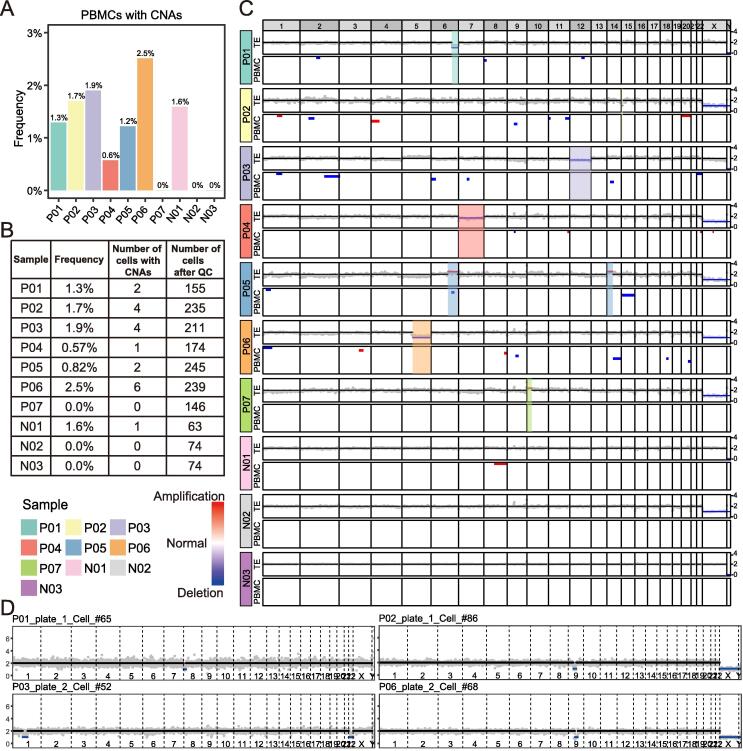
**Profiles of abnormal cells in peripheral blood of healthy live infants from the transfer of mosaicism embryos and normal embryos** **A.** The fraction of PBMCs with CNAs in each infant. **B.** Number of PBMCs that passed QC and PBMCs with CNAs in each infant. **C.** The TE PGT-A results and CNA profiles of PBMCs from 10 infants. Each column represents a genomic window of approximate 1.0 Mb. For each infant, the dot plot (above) shows the normalized read count (gray) and inferred copy number (black for normal, blue for deletion, and red for amplification) of each genomic window in TE PGT-A; the heatmap (below) shows copy number profiles of 10 selected PBMCs, with a preference for PBMCs with CNAs (white for normal, blue for deletion, and red for amplification). Note that for males, having one copy of the X and the Y chromosome in each cell is normal. **D.** Copy number profiles of four examples of PBMCs with CNAs from four infants. The gray point shows the normalized read count of each 1.0 Mb window. The black, red, and blue horizon lines represent normal, amplification, and deletion, respectively. CNA, copy number alteration; QC, quality control.

We examined seven infants with embryonic chromosomal mosaicism and three infants with euploid TE biopsy results as the controls, with a total of 2208 cells (200–300 cells per “mosaic” infant) being sequenced and a total of 1616 cells passing our strict quality control (Tables S1 and 2). The results showed that some cells of both the “mosaic” and control infants have CNAs (> 10 Mb), varying between 0%−2.5% (Figure 2A and B). These somatic CNAs in blood cells showed relatively random patterns and seemed to be non-clonal, occurring at an average frequency of 1.2%. The presence of these somatic CNAs has been reported recently [18], and it seems that the somatic CNA frequency was lower in these infants compared with that in adults [18] (Figure 2A–C; Table S3). The normal euploid cells were clearly distinguished from abnormal aneuploid cells (Figure 2D).

Next, we focused on the regions where the TE biopsies have shown abnormal CNAs. The results showed that the vast majority of the single cells are normal in copy number in these regions (Figure 2C). One exception was a cell from case P05, which showed partial deletion in the abnormal region in the TE biopsy; however, the length of deletion was much shorter than that in the original region, and the ploidy change was opposite-gained copies in TE biopsies, whereas lost copies in peripheral blood mononuclear cells (PBMCs) were detected (Figure 2C). We concluded that none of the analyzed cells displayed a gain or loss of CNAs corresponding to that of the corresponding TE biopsy (Figure 2C).

In summary, we found that the infant’s peripheral blood cells had a lower proportion of cells containing CNAs, and these CNA events were different from the CNA events in the previous TE biopsy results, showing relatively random distributions.

### Characteristics of cell identity in mosaic blastocyst transplanted infants

To further confirm the cell identity of single cell sorted by FACS, we analyzed matched transcriptome information in single-cell multi-omics sequencing data. And a total of 988 cells passed strict quality control (Figure S1A and B; Table S2), dividing them into 6 major cell types (T cells, natural killer cells, B cells, monocytes, dendritic cells, and unclassified) according to known markers (Figure S1C–E). The number of different cell types captured varies from individual to individual (Figure S1F), depending on sampling time and mode of transport. Among the cells we analyzed, a total of 20 cells had very clear CNAs. Combined with transcriptional information, it was found that these abnormal cells were distributed in a variety of blood cell types (Figure S1G and H).

In a word, using single-cell transcriptome data, we found that the cells with CNAs were of various blood cell types.

## Discussion

Among the 1616 blood cells obtained from seven infants, none showed a gain or loss of the same chromosome regions as in the TE biopsy. These results suggested that embryonic chromosomal mosaicism of the infants was corrected by some mechanisms, probably through the selective loss of the aneuploid cells during development. And the relatively low percentages of the aneuploid cells in the embryos at the blastocyst stage seem not to affect the growth and development of the individual. As for how embryos with chromosomal abnormalities produce healthy babies, we think there may be two possible scenarios. First, the embryo has a self-correcting mechanism. Most aneuploidies arise from maternal meiosis, and they increase exponentially in women over the age of 35 years [19], [20]. These meiotic aneuploidies usually affect every cell in an embryo and will not generate aneuploid–euploid mosaic embryos. Mitotic errors are also very common in early embryo development [21], which often include chromosome nondisjunction, premature cell division before DNA replication, and inadvertent chromosome destruction [6], [22]. These mitotic aneuploidies usually affect only some of the cells in an embryo and will generate aneuploid–euploid mosaic embryos. Studies have shown that the aneuploid cells from the inner cell mass (ICM) lineage in mouse blastocysts are selectively eliminated by apoptosis, while the aneuploid cells from the TE lineage show serious proliferation defects, and the proportion of aneuploid cells gradually decreases from the blastocyst stage [23]. Another study has reported that the proliferation and death dynamics of euploid and aneuploid cells in early embryos are different. Aneuploid cells proliferate slower and will undergo apoptosis, while euploid cells proliferate faster and can compensate for cell loss in a mosaic embryo [24]. Second, TE biopsy results are not fully representative of ICM in the same blastocyst due to the potentially different percentages of aneuploid cells between TE and ICM. However, we have not explored how mosaic embryos become completely normal infants, which needs to be studied in the future.

During the preparation of our manuscript, Capalbo et al. [25] conducted a prospective study, and they found that blastocysts with a mosaic biopsy specimen have similar developmental potential to blastocysts with an entirely euploid TE biopsy sample. Postnatal genotyping was also performed on some of the infants born from the mosaic embryo transfer, and the results showed a completely normal karyotype and no uniparental ditype. They used single-nucleotide polymorphic array (SNPa) to test newborn saliva samples in bulk and confirmed that the newborns did not have abnormal karyotypes in the ectoderm-derived cells. In our study, we performed both verifications of ploidy and identity characteristics in a single cell with high resolution. More than 200 cells were analyzed in each “mosaic” infant. Even just one individual cell had CNAs at the same position as the original mosaicism, our technology still could accurately detect it, which gave higher sensitivity than the method used by Capalbo and colleagues [25]. Among the 1616 examined cells, we did not find any cells with CNAs at the original mosaicism site, indicating that mosaicism disappeared (or decreased to at least lower than 1%) at least in the hemopoietic lineage in these cases. In addition to peripheral lymphocytes, we also attempted to analyze the buccal cells, but these cells were exfoliated dead cells and could not pass the strict quality control of single-cell multi-omics sequencing data analysis. It is very difficult to test the infant’s cells of other tissues because it is difficult to get permission to perform such invasive sampling. Our study complemented that of Capalbo et al. [25] and together indicated that transplanted mosaic embryos could produce healthy babies with normal ploidy.

At the same time, we also note that Kahraman et al. [26] reported a case of female birth with chromosome 2 mosaicism. The female baby showed 35% monosomy on chromosome 2 at the blastocyst stage and 2% trisomy on chromosome 2 during amniocentesis. After birth, peripheral blood analysis revealed a 2% mosaic monosomy on chromosome 2. Although the newborn had no significant phenotype and was normal during development and growth, this case highlights the need for careful genetic counseling, particularly prenatal monitoring and diagnosis, in patients who choose mosaic embryo transfer [27], [28], [29]. It is necessary to establish a unified standard for mosaic embryo transfer and monitoring.

## Conclusion

Our study provides new insights into the consequence of TE-biopsy-diagnosed mosaic embryos, suggesting that at least a portion of these embryos can successfully develop into healthy infants with full correction of the chromosomal abnormality, probably through the selective loss of the aneuploid cells during development, and supporting mosaic embryo transfer in certain conditions. This offers new hope for patients with poor ovarian reserve, a limited number of embryos, or only embryos with chromosomal abnormalities detected, which represent the majority of IVF patients.

## Materials and methods

### Collection of clinical samples

We collected peripheral blood samples from 10 infants, all from IVF embryos, and biopsied TE during their blastocyst stage. Biopsies revealed mosaic chromosomes in 7 infants and completely normal chromosomes in 3 infants. All of these babies were born normally. Sometime after they were born, we monitored their health and collected trace amounts of blood from their fingertips for multi-omics library construction. Trace blood was stored in a culture medium containing advanced DMEM/F12 medium (Catalog No. 12634-010, ThermoFisher Scientific, Waltham, MA), 1× GlutaMAX (Catalog No. 35050-061, ThermoFisher Scientific), 1× penicillin–streptomycin (Catalog No. 15140-122, ThermoFisher Scientific), 10 mM 2-[4-(2-hydroxyethyl)piperazin-1-yl]ethanesulfonic acid (HEPES; Catalog No. 15630-080, ThermoFisher Scientific), and 8 mM ethylene diamine tetraacetic acid (EDTA; Catalog No. 15575-020, ThermoFisher Scientific). For samples outside Beijing, low-temperature transportation of 4 ^o^C is adopted.

### Sample handling

We treated peripheral blood with CD45 antibody (Catalog No. 368510, BioLegend, San Diego, CA) for 20 min on ice. Then red blood cells were lysed with lysis buffer (Catalog No. R7757-100ML, Sigma-Aldrich, Saint Louis, MO) for 5 min. The remaining cells were collected by centrifugation and then resuspended with 0.1% bovine serum albumin (BSA; Catalog No. 37525, ThermoFisher Scientific) in Dulbecco’s phosphate-buffered saline (DPBS; Catalog No. D8537-500ML, Sigma-Aldrich). And 7-AAD viability staining solution (Catalog No. 420403, BioLegend) was added to exclude nonviable cells. We sorted single CD45^+^7-AAD^−^ immune cells into 96-well plates for the following steps by BD FACS ARIA SORP (BD Biosciences, Franklin Lakes, NJ).

### Parallel single-cell genome and transcriptome sequencing

Single-cell multi-omics sequencing libraries were prepared according to the previously published method, and the accuracy and sensitivity of the method have been systematically evaluated [18]. Briefly, nuclear fractions of 96 mouse embryonic stem cells were pooled together and evenly split into 96 cells as 96 “average cells” to eliminate the potential low frequency of CNAs. No CNAs were detected in any “average cells”, confirming that our method is very accurate in detecting CNAs. We used this method to conduct subsequent experiments using infant peripheral blood cells. In brief, single cells were sorted into 96-well plates with 3 μl lysis buffer, which contains Dynabeads Myone carboxylic acid (Catalog No. 65011, ThermoFisher Scientific), RNase inhibitor (Catalog No. 2313A, Takara, Japan), Triton X-100 (Catalog No. T9284, Sigma-Aldrich), and dNTP (Catalog No. 4019, Takara). We vortexed the plates to lyse cells sufficiently and then put them on magnetic racks to separate the mRNAs from the nucleus. The supernatants containing mRNAs were transferred into the new 96-well plates. SuperScript II reverse transcriptase (Catalog No. 18064071, ThermoFisher Scientific) and barcoded-polyT-RT primers were used to perform reverse transcription. Then pooled complementary DNAs (cDNAs) were amplified by KAPA HiFi HotStart ReadyMix (Catalog No. KK2602, KAPA Biosystems, Boston, MA). Cell nuclei aggregated with beads were resuspended with multiple annealing and looping-based amplification cycle (MALBAC) lysis buffer to protease digestion. Genomic DNAs (gDNAs) were amplificated for 11 cycles of quasilinear pre-amplification with DeepVent (exo-) DNA polymerase (Catalog No. M0259L, New England BioLabs, Ipswich, MA), GAT-N5-3G primers, and GAT-N5-3T primers. Then 12 cycles of exponential amplification were carried out with DeepVent (exo-) DNA polymerase and barcode-GAT primers.

We pooled cDNAs and gDNAs with different barcodes together and purified them for the biotin-amplification with biotin-modified primers. Then, cDNA and gDNA products were sheared into approximately 300 bp by M220 focused-ultrasonicator (Covaris, Woburn, MA) and captured by Dynabeads Myone streptavidin C1 (Catalog No. 65001, ThermoFisher Scientific). The libraries were generated with KAPA Hyper Prep Kit (Catalog No. KK8505, KAPA Biosystems) and sequenced for 150 bp paired-end reads on Illumina NovaSeq 6000 platform (Illumina, San Diego, CA).

### Processing single-cell genome sequencing data

Cell barcodes (8 nt) and primer (35 nt) in the reads 2 of paired-end sequencing reads were extracted and added to the sequence identifier of corresponding reads 1 by unique molecular identifiers (UMI) tools (version 1.0.1) [30]. Adaptor sequence and low-quality reads were then trimmed by fastp (version 0.20.1) [31] with default parameters. The filtered reads were mapped to the human reference genome (hg19) using BWA (version 0.7.17) [32]. Mapped reads with mapping quality (MAPQ) ≥ 30 were retained for downstream analysis. BAM files were sorted and indexed using SAMtools (version 1.11) [33]. Polymerase chain reaction (PCR) duplicates were identified and removed by Picard (version 2.19.0) (https://broadinstitute.github.io/picard/). The BAM files were converted to BED files using the bamToBed command of BEDTools (version 2.24.0) [34]. The BED files were then demultiplexed according to the cell-specific barcodes in sequence identifier.

### Estimation of single-cell CNAs from single-cell genome sequencing data

Single-cell CNAs were estimated as described in a previous study [35]. The genome was tiled into 2808 variable-sized bins with an approximate length of 1.0 Mb. Predefined ‘bad bins’ were excluded from further processing. Read counts in each bin were normalized by sequence depth of the cell and then corrected for GC content using LOWESS regression. Cells from the same 96-well plates were pooled together as a control to eliminate amplification bias. Circular binary segmentation (CBS) was used to segment the read count profile of each cell, with the parameters ‘alpha = 1E−10’ and ‘undo.prune = 0.05’. Copy number of each segment was then determined using numerical optimization. For each cell, the coefficient of variation (CV) of read count in each bin was calculated. Cells with CV ≥ 1 or total usable unique read number ≤ 3E−5 were excluded. Remaining cells were further filtered for CNA profiles by an additional manual check. In total, 1616 of 2208 cells (73.2%) passed quality control and were retained in downstream analysis. Only cells with CNAs longer than 10 Mb and confirmed to be of good quality by manual check were considered to have CNAs. For female cells, the Y chromosome was not considered.

### Processing single-cell RNA sequencing data

Reads which cannot perfectly match with the cell barcode (8 nt) used were removed. Cell barcodes and UMIs (8 nt) in the reads 2 of paired-end sequencing reads were extracted and added to the sequence identifier of corresponding reads 1 by UMI-tools (version 0.5.4) [30] with the parameter ‘--bc-pattern = CCCCCCCCNNNNNNNN’. Template switch oligo (TSO) sequence and polyA sequence were trimmed by a custom Perl script. Adaptor sequence and low-quality reads were filtered by fastp (version 0.20.1) [31]. The clean reads were mapped to the human reference genome (hg19) using STAR (version 2.6.0a) [36]. Aligned reads were annotated to RefSeq genes using featureCount of the subread package (version 1.6.2) [37]. The BAM files were then sorted and indexed using SAMtools (version 1.7) [33]. The number of UMIs assigned to each gene was counted using UMI-tools (version 0.5.4) [30] with the parameter ‘--per-gene --gene-tag = XT -per-cell --wide-format-cell-counts’. Cells with less than 300 detected genes or more than 30 percent mitochondrial reads were excluded.

### Pre-implantation genetic testing

IVF, embryo culture, and blastocyst biopsy were performed as previously described [38]. NGS-based PGT for aneuploidy (PGT-A) was performed in the molecular laboratory using pre-implantation genetic screening for aneuploidy kit (Catalog No. R0009, Berry Genomics, Beijing, China) according to the manufacturer’s protocol. Copy number variation sequencing (CNV-seq) was carried out as previously described, which is a well-established technology and has been widely used for prenatal testing and screening for chromosome disease syndromes [39], [40]. In brief, blastocyst embryo biopsy samples were lysed, and gDNAs were fragmented to 300 bp. Random primers were added for pre-amplification, and then universal primers for high-throughput sequencing and index primer for different samples were added for amplification to complete the construction of the sequencing library. Libraries were sequenced using the NextSeq CN500 platform (Illumina).

### Processing of CNV-seq data from TE biopsy

Sample-specific barcodes (7 nt) were extracted and added to the sequence identifier of single-end reads using UMI-tools (version 1.0.1) [30]. Clean reads were mapped to the human reference genome (hg19) using BWA (version 0.7.17) [32]. MAPQ ≥ 30 were retained for downstream analysis. BAM files were sorted and indexed using SAMtools (version 1.11) [33]. PCR duplicates were identified and removed by Picard (version 2.19.0; https://broadinstitute.github.io/picard/). The BAM files were converted to BED files using the bamToBed command of BEDTools (version 2.24.0) [34].

CNAs in TE biopsy were estimated using similar methods as used in single cells, with a few modifications. The genome was tiled into 1048 variable-sized bins with an approximate length of 2.5 Mb. After the first round of CNA estimation, samples with no CNAs and confirmed to be of good quality by manual check were pooled together as a control for the second round of CNA estimation to eliminate amplification bias.

## Ethical statement

This study was approved by the medical ethics committee of the Chinese PLA General Hospital (Approval No. S2015-058-02). Samples were collected with the informed consent of the infant’s parents.

## Code availability

The source code has been submitted to BioCode, and is available at https://ngdc.cncb.ac.cn/biocode/tools/BT007311. It is also available on the GitHub website (https://github.com/liuzhenyu-yyy/IVF_Mosaic_Embryo_Transfer).

## Data availability

All sequencing data of this study have been deposited in the Genome Sequence Archive [41] at the National Genomics Data Center, Beijing Institute of Genomics, Chinese Academy of Sciences / China National Center for Bioinformation (GSA: HRA001848), and are publicly accessible at https://ngdc.cncb.ac.cn/gsa-human/.

## CRediT author statement

**Yuan Gao:** Conceptualization, Methodology, Validation, Investigation, Writing - original draft, Writing - review & editing, Visualization, Project administration. **Jinning Zhang:** Validation, Investigation, Resources, Writing - review & editing. **Zhenyu Liu:** Software, Validation, Formal analysis, Data curation, Writing - review & editing, Visualization. **Shuyue Qi:** Methodology, Validation, Investigation, Writing - review & editing. **Xinmeng Guo:** Validation, Resources. **Hui Wang:** Resources. **Yanfei Cheng:** Resources. **Shuang Tian:** Resources. **Minyue Ma:** Resources. **Hongmei Peng:** Resources. **Lu Wen:** Conceptualization, Writing - review & editing, Supervision, Funding acquisition. **Fuchou Tang:** Conceptualization, Validation, Writing - review & editing, Supervision, Project administration, Funding acquisition. **Yuanqing Yao:** Conceptualization, Validation, Resources, Supervision, Funding acquisition. All authors have read and approved the final manuscript.

## Competing interests

The authors declare they have no competing interests.

## Supplementary material

Supplementary Figure 1 Transcriptome aspects of single-cell multi-omics sequencing confirm that CNAs are detected in different cell types **A.** Number of UMIs and detected genes for cells of different cell types. **B.** Quality control of single-cell RNA-Seq. Cells with more than 300 detected genes and less than 30% mitochondrial reads are retained for downstream analysis. 988 cells passed quality control. **C.** UMAP plot of all 988 cells passed RNA quality control. Cells are colored by subjects. **D.** UMAP of selected known marker genes to identify PBMC cell type. **E.** Violin plot showing the expression levels of known marker genes in each cell type. *PTPRC* indicates immune cells; *CD3D* and *CD3G* indicate T cells; *NKG7* and *KLRF1* indicate NK cells; *MS4A1* and *CD79A* indicate B cells; *CD14* and *LYZ* indicate *CD14+* monocytes; *FCGR3A* and *MS4A7* indicate *FCGR3A+* monocytes; *CST3* and *ITGAX* indicate dendritic cells. **F.** Fraction of each cell type in each subject. **G.** UMAP plot shows the cell type of the cells. **H.** UMAP plot shows the cells with CNAs. Normal cells are colored in light gray. Cells with CNAs are colored by cell types. UMIs, unique molecular identifiers; NK, natural killer cell; DC, dendritic cell; UMAP, uniform manifold approximation and projection.

Supplementary Table S1 **Infant information statistics**

Supplementary Table S2 **Summary of sequenced cells**

Supplementary Table S3 **Summary of cells with CNAs**
