# Supplementary figures and images for: Single-cell Sequencing Reveals Clearance of Blastula Chromosomal Mosaicism in In Vitro Fertilization Babies
Source: Genomics Proteomics Bioinformatics. 2022 Aug 6;20(6):1224–31. doi: 10.1016/j.gpb.2022.07.004 (PMC10225483; doi:10.1016/j.gpb.2022.07.004)

Fig.S1

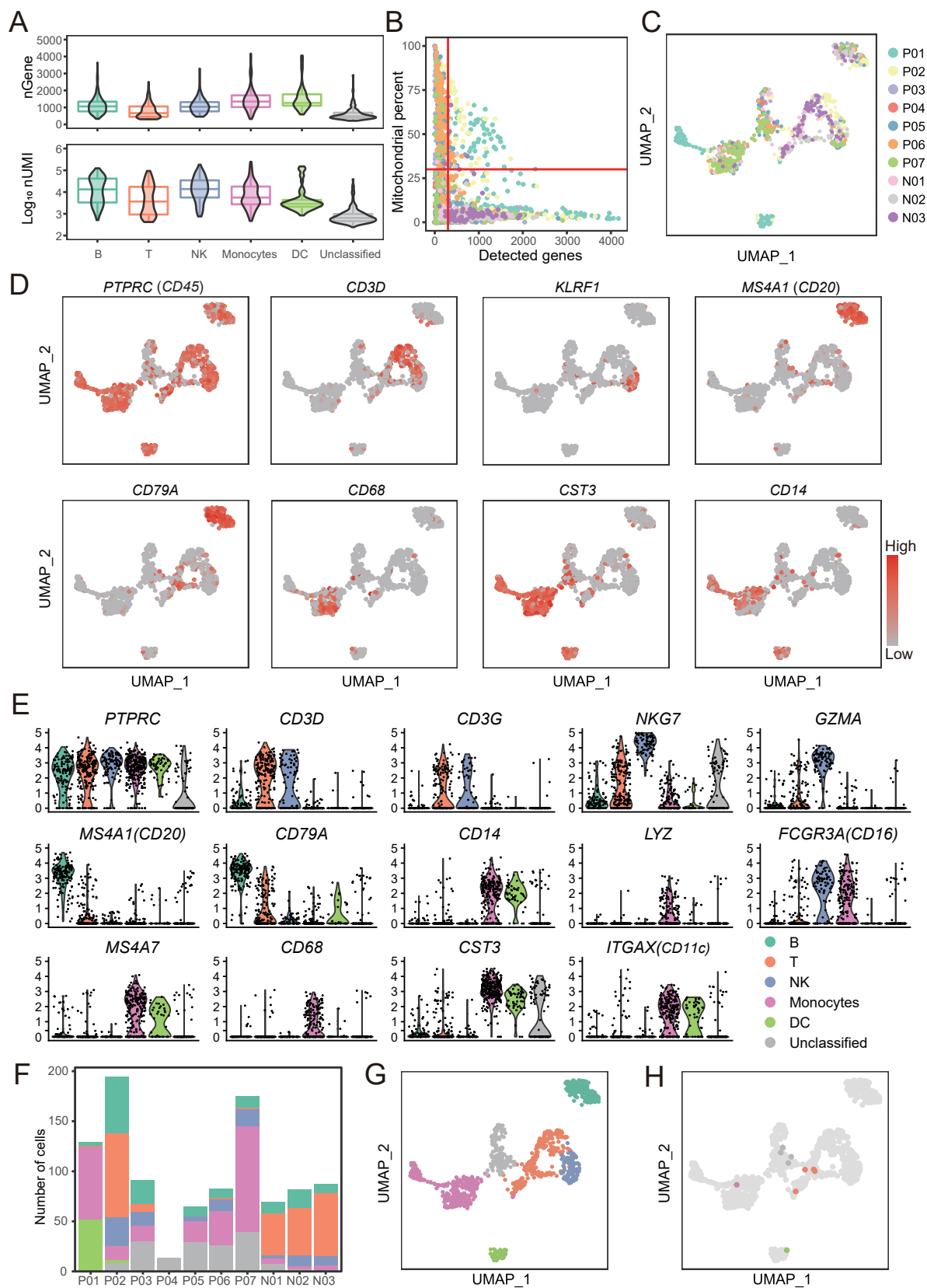

Supplement: Supplementary Figure 1 — Transcriptome aspects of single-cell multi-omics sequencing confirm that CNAs are detected in different cell types A. Number of UMIs and detected genes for cells of different cell types. B. Quality control of single-cell RNA-Seq. Cells with more than 300 detected genes and less than 30% mitochondrial reads are retained for downstream analysis. 988 cells passed quality control. C. UMAP plot of all 988 cells passed RNA quality control. Cells are colored by subjects. D. UMAP of selected known marker genes to identify PBMC cell type. E. Violin plot showing the expression levels of known marker genes in each cell type. PTPRC indicates immune cells; CD3D and CD3G indicate T cells; NKG7 and KLRF1 indicate NK cells; MS4A1 and CD79A indicate B cells; CD14 and LYZ indicate CD14+ monocytes; FCGR3A and MS4A7 indicate FCGR3A+ monocytes; CST3 and ITGAX indicate dendritic cells. F. Fraction of each cell type in each subject. G. UMAP plot shows the cell type of the cells. H. UMAP plot shows the cells with CNAs. Normal cells are colored in light gray. Cells with CNAs are colored by cell types. UMIs, unique molecular identifiers; NK, natural killer cell; DC, dendritic cell; UMAP, uniform manifold approximation and projection. [file mmc1.pdf]
